# Liver dysfunction triggers early Alzheimer’s pathology in an adult rat model of chronic liver disease

**DOI:** 10.1038/s41598-025-21054-x

**Published:** 2025-10-30

**Authors:** O. Braissant, V. A. McLin, D. Sessa, K. Pierzchala

**Affiliations:** 1 Service of Clinical Chemistry, Lausanne University Hospital and University of Lausanne, Lausanne, Switzerland; 2 Department of Pediatrics, Gynecology and Obstetrics, Swiss Pediatric Liver Center, University Hospitals Geneva and University of Geneva, Geneva, Switzerland; 3 CIBM Center for Biomedical Imaging, EPFL AVP CP CIBM-AIT, CH F1 602, Station 6, 1015 Lausanne, Switzerland; 4 CIBM-PCI EPFL Metabolic Imaging Section, Ecole Polytechnique Fédérale de Lausanne, Lausanne, Switzerland

**Keywords:** Chronic liver disease, Alzheimer’s disease, Neurodegeneration, Neurometabolism, Neurofilaments, Amyloid-β, Tau-bodies, Biomarkers, Liver diseases, Comorbidities, Neuroscience, Alzheimer's disease, Neurodegeneration

## Abstract

Emerging evidence links liver dysfunction to Alzheimer’s disease (AD), though few studies have investigated this connection. While cirrhosis is associated with cognitive impairment, its underlying mechanisms remain poorly understood. This study aimed to assess the presence of central nervous system (CNS) markers of Alzheimer’s disease in a rat model of chronic liver disease. Standard histochemical techniques were employed, including Congo red staining for amyloid-β (Aβ) and Gallyas silver staining for tau pathology. Immunohistochemistry was used to evaluate changes in aquaporin (Aqp1, Aqp4, Aqp9) expression and astrocytic glial fibrillary acidic protein (GFAP) levels. Furthermore, blood concentrations of neurodegeneration markers, neurofilament light chain (NfL), Aβ, phosphorylated tau (p-tau), total tau (t-tau), GFAP, and myelin oligodendrocyte glycoprotein (MOG), along with bile acids, were quantified and compared between BDL and SHAM control groups to investigate potential systemic correlates of CNS pathology. CNS analysis revealed the presence of intracellular amyloid-β (iAβ) and abnormal tau aggregates (pre-tangle/tangle stages), consistent with early AD pathology. Additionally, alterations in aquaporin water channels and astrocytic GFAP expression were observed. Peripheral blood analysis showed significant changes in neurodegeneration markers (NfL, Aβ, p-tau, t-tau, GFAP, MOG) and bile acid profiles, reinforcing the systemic nature of neuroinflammatory processes in liver disease. Our findings emphasize liver failure as a significant factor in cognitive decline and increased AD risk. The study advocates for incorporating liver function screening into the diagnostic workup of patients with dementia.

**Supplementary Information:**

The online version contains supplementary material available at 10.1038/s41598-025-21054-x.

## Introduction

Every year, over 10 million new cases of dementia are diagnosed worldwide^1^. Alzheimer’s disease (AD) accounts for 60–80% of these cases and primarily manifests as memory loss and cognitive decline. Additionally, approximately 5–10% of individuals develop vascular dementia^2^. The brain changes associated with AD are believed to begin 20 years or more before symptoms, involving glucose hypometabolism, amyloid β (Aβ) accumulation, tau protein hyperphosphorylation (resulting in neurofibrillary tangles formation (NFTs)), neurodegeneration and neuroinflammation^3^.

Growing evidence highlights liver role in AD pathogenesis. Few studies have explored this link^4,5^, some suggesting an impairment of Aβ clearance by liver^6^. Studies confirm association between cirrhosis and cognitive impairment^7,8^, suggesting liver disfunction as an important risk factor causing cognitive decline^7,8^. There is increasing interest in the role of liver dysfunction as a potential contributor to Aβ deposits^4,9^.

Despite progress in understanding the mechanisms of type C hepatic encephalopathy (HE), a consequence of chronic liver disease (CLD) affecting up to 80% of patients, the impact on cognition and dementia risk remains poorly defined^10–12^. Cognitive dysfunction in type C HE involves attention and memory deficits^13^. This raises the question of whether these neurocognitive impairments are linked to AD—an area that remains underexplored. The association between hyperammonemia and cognitive decline has been discussed in the literature since the early 1980s^14^. At that time, studies showed that Alzheimer’s patients were unable to maintain ammonia levels within the normal range^14^. Ammonia, a recognized neurotoxin, may contribute to AD progression. Unfortunately, the role of the gut-liver-brain axis in neuropathology has only recently regained scientific attention, highlighting the importance of liver health for central nervous system (CNS) function^4,8,15^.

It is also well established that neurodegenerative diseases cause metabolic and cellular abnormalities in the brain. We have previously demonstrated neurometabolic changes in BDL rat model (bile duct ligation (BDL))^16^ of CLD including increased glutamine levels and alterations in neurotransmitters, osmolytes, antioxidants, and glucose metabolism^11,17,18^. These alterations were accompanied by increased oxidative stress (OS) and inflammation, both systemic and within the CNS, notably characterized by a significant accumulation of IL-6 in the brain, a pleiotropic pro-inflammatory cytokine ^10,12^. Furthermore, increased IL-6 concentrations observed in the brains of BDL rats have been associated with aging processes, central nervous system injury responses, compromised blood–brain barrier integrity, and deterioration of memory and cognitive performance^10^. Moreover, our recent studies in BDL rats revealed significant morphological activation-related changes in astrocytes and microglia, along with morphological alterations in neurons^11,17,19^.

**Fig. 1:**
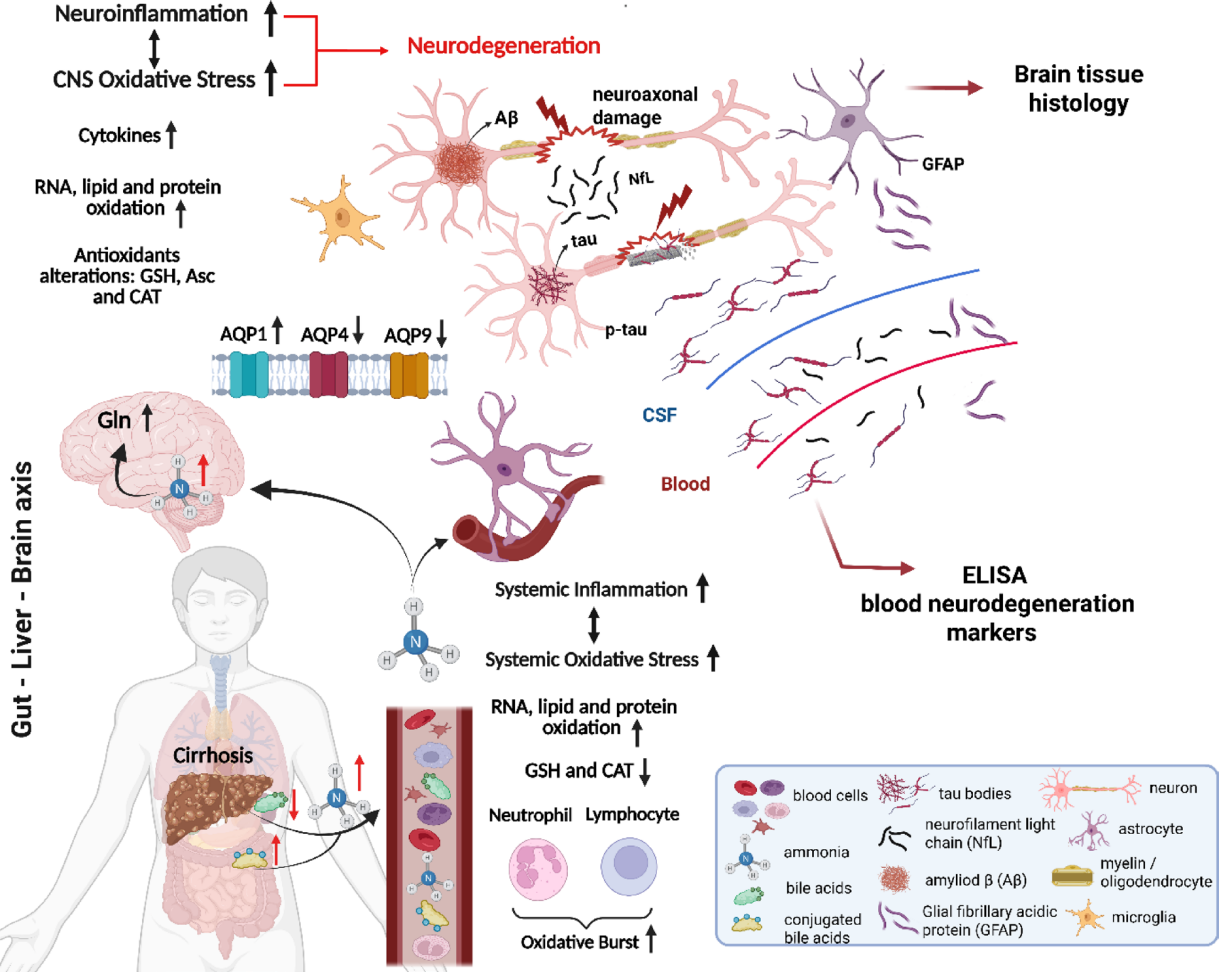
**Overview of existing knowledge and study insights.** Created with BioRender.com.

To the best of our knowledge, this is the first longitudinal study analyzing the Alzheimer’s disease-related features in a BDL rat model, an established model of CLD (Fig. 1).

Our integrated approach uniquely combines the experimental advantages of CNS histochemistry, immunohistochemistry, and UV–Vis spectroscopy of immunolabeled brain sections, alongside the detection of blood-based (systemic) neurodegeneration markers (neurofilament light chain (NfL), Aβ, phosphorylated tau (p-tau), total tau (t-tau), glial fibrillary acidic protein (GFAP), and myelin oligodendrocyte glycoprotein (MOG)), provides a comprehensive view of disease progression from peripheral to central levels.

## Results

### Chronic liver disease induction

**Fig. 2:**
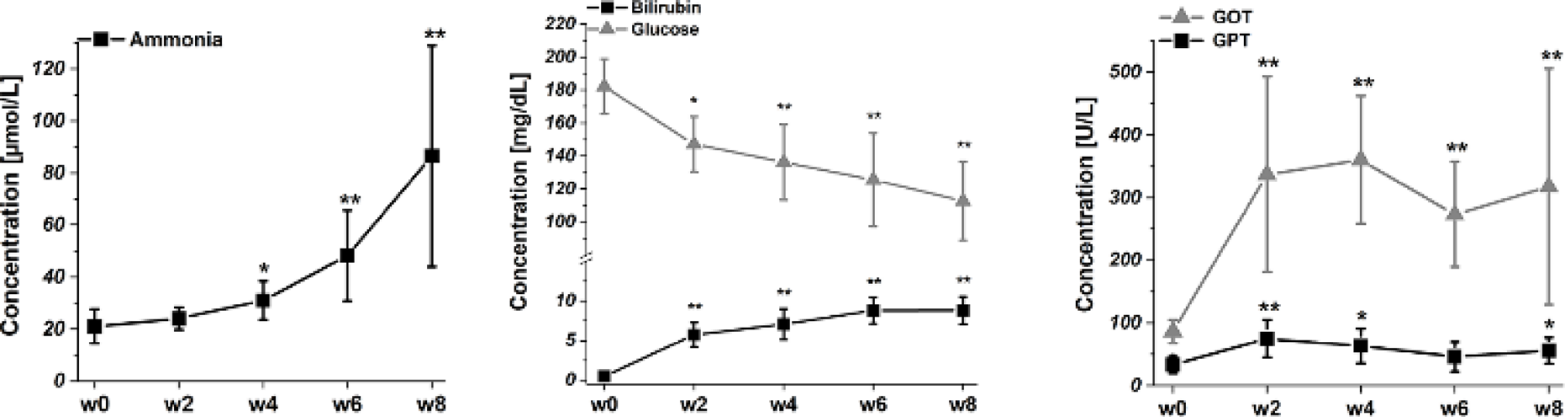
**Longitudinal changes in blood ammonia, total plasma bilirubin, glucose, GOT and GPT induced by the bile duct ligation.** Bilirubin was undetectable before BDL. Data are presented as mean ± SD and statistical significance between week 0 and weeks 2–8: *p < 0.05, **p < 0.01 (One-way Anova with post-hoc Tukey HSD).

Blood biochemistry confirmed chronic liver disease (CLD). In line with our previous findings^11,12,19^, we observed an early (2 weeks post-BDL) increase in blood ammonia, plasma bilirubin, and liver function markers, such as aspartate aminotransferase (AST/GOT) and alanine aminotransferase (ALT/GPT) (Fig. 2). Additionally, a significant decline in blood glucose levels was noted as the disease progressed (Fig. 2).

### Histology

#### Histochemistry: signatures of Alzheimer disease in CLD

**Fig. 3:**
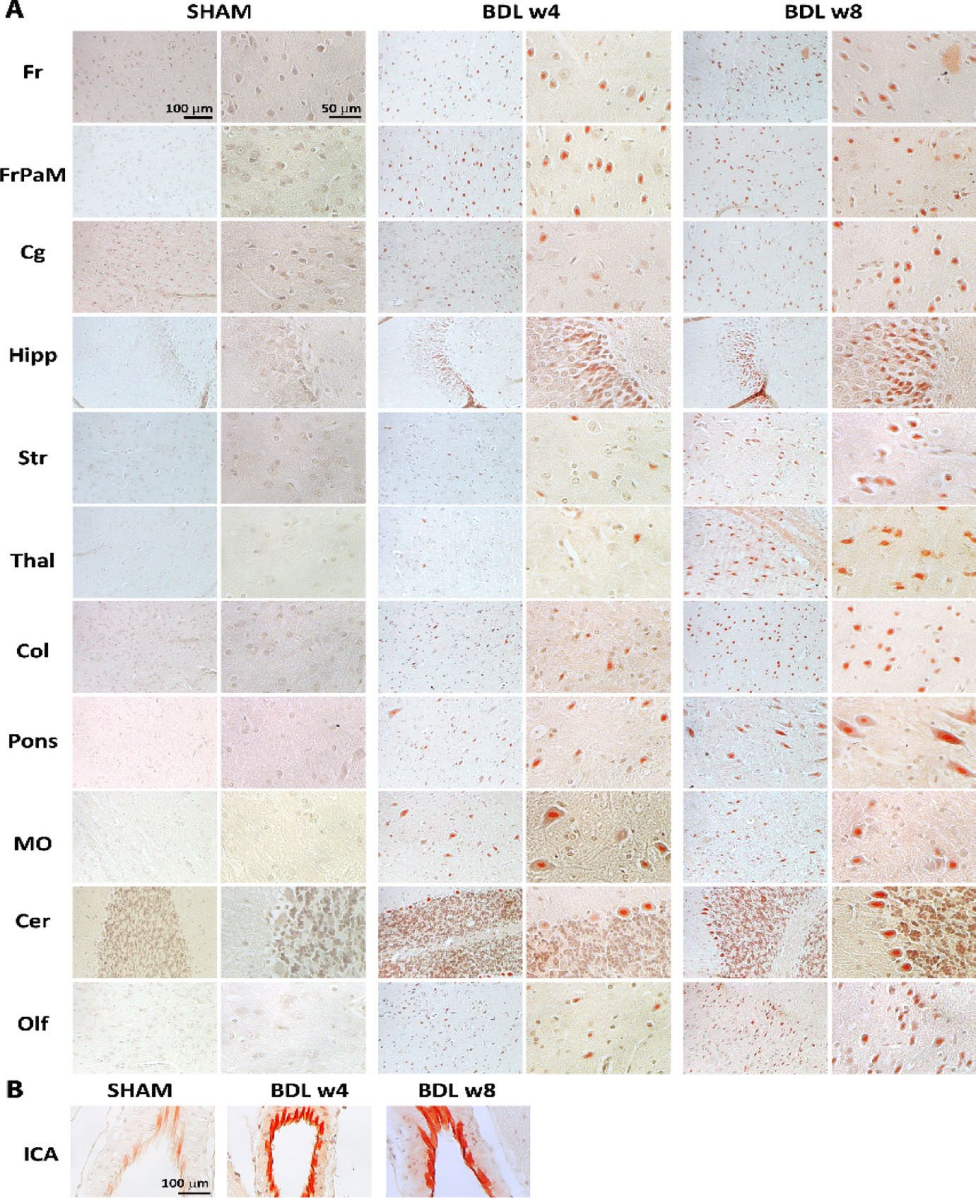
**Amyloid β accumulation in the brain visualized with Congo red histochemical stain.** Representative microphotographs of the BDL rats’ brain and age-matched SHAM controls. Initial intraneuronal Aβ deposits (red) are detectable at 4-weeks post-BDL, and increase with disease progression. The BDL revealed positive signal of intracellular Aβ accumulation in frontal cortex (Fr), frontoparietal cortex—motor area (FrPaM), cingulate gyrus (Cg), hippocampus (Hipp), striatum (Str), thalamus (Thal), midbrain colliculus (Col), Pons, medulla oblongata (MO), cerebellum (Cer), olfactory tubercle (Olf) (**A**), and also deposits in brain arteries, here the internal common carotid artery (ICA) (**B**) indicating cerebral amyloid angiopathy.

At 4-weeks post BDL surgery, Congo red staining revealed intracellular Aβ accumulation in several brain regions of BDL rats, with the accumulation increasing as the disease progressed in frontal cortex (Fr), frontoparietal cortex—motor area (FrPaM), cingulate gyrus (Cg), hippocampus (Hipp), striatum (Str), thalamus (Thal), midbrain colliculus (Col), Pons, medulla oblongata (MO), cerebellum (Cer), olfactory tubercle (Olf) (Fig. 3A). In addition, there was a strong accumulation of Aβ in the region of neuronal nuclei and diffuse Aβ in the cytoplasm (Fig. 3A, Fig. S1). In addition, abnormalities of the arterial vessel wall suggested (Fig. 3B, Fig. S1) cerebral amyloid angiopathy (CAA).

**Fig. 4:**
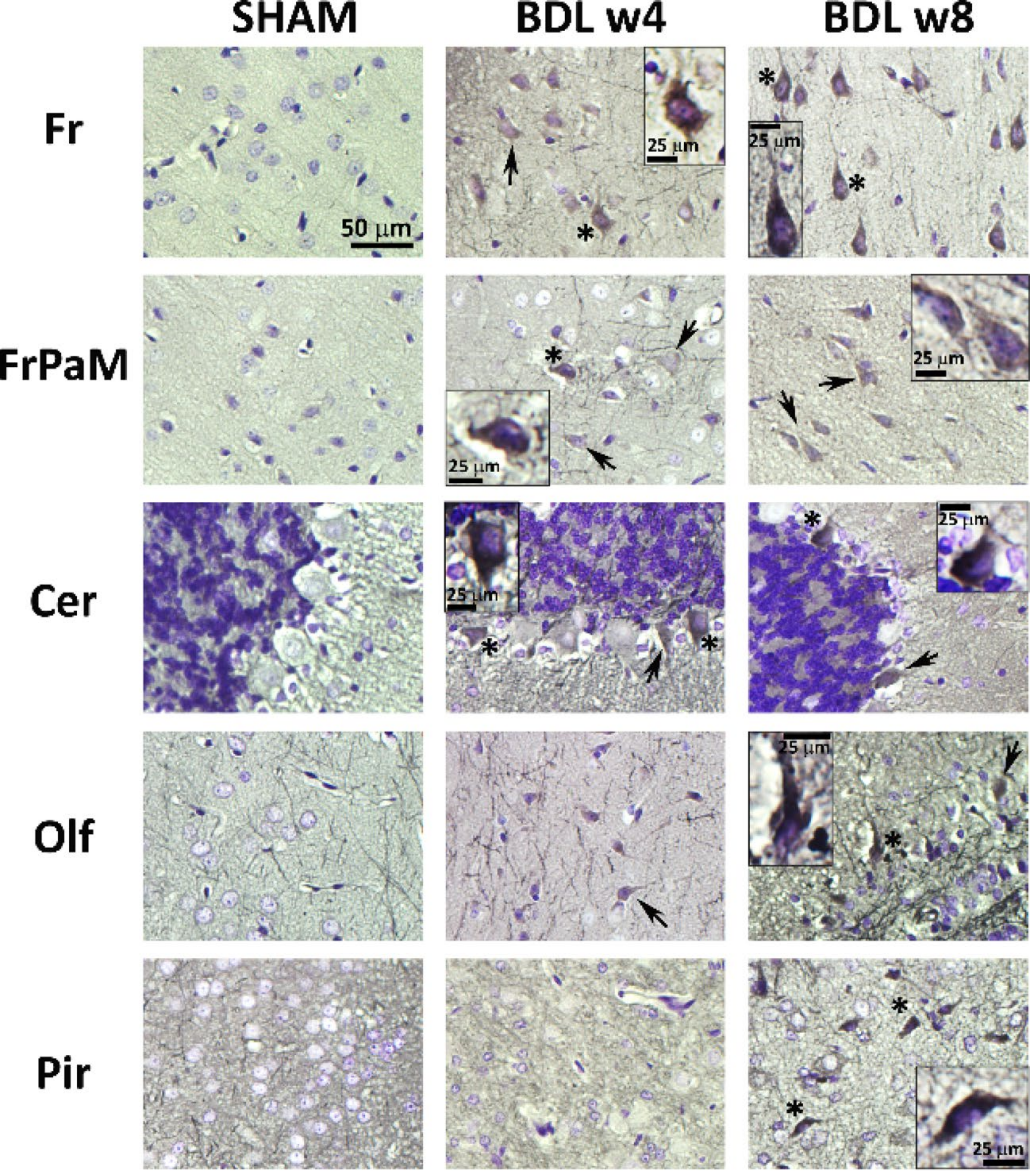
**Representative micrographs of Gallyas silver stain **of the BDL rat’s brain and age-matched SHAM controls revealed accumulation of abnormal tau-protein in the pre-tangle status (arrow) and more advanced forms of NFTs (asterisk) in frontal cortex (Fr), frontoparietal cortex—motor area (FrPaM), cerebellum (Cer), olfactory tubercle (Olf) and piriform cortex (Pir).

Gallyas staining revealed abnormal tau-bodies in the pre-tangle and neurofibrillary tangles (NFTs) status in the frontal cortex (Fr), frontoparietal cortex—motor area (FrPaM), cerebellum (Cer), olfactory tubercle (Olf) and piriform cortex (Pir) (Fig. 4).

#### Immunohistochemistry and immunofluorescence (UV–Vis spectroscopy)

 Aqp1, Aqp4, Aqp9 and GFAP staining analysis was performed in the hippocampus and cerebellum. Immunolabeling followed by UV–Vis spectroscopy of immunofluorescence revealed a statistically significant fluorescence signal intensity alterations in all the labeled proteins already at 4-weeks post-BDL.

**Fig. 5:**
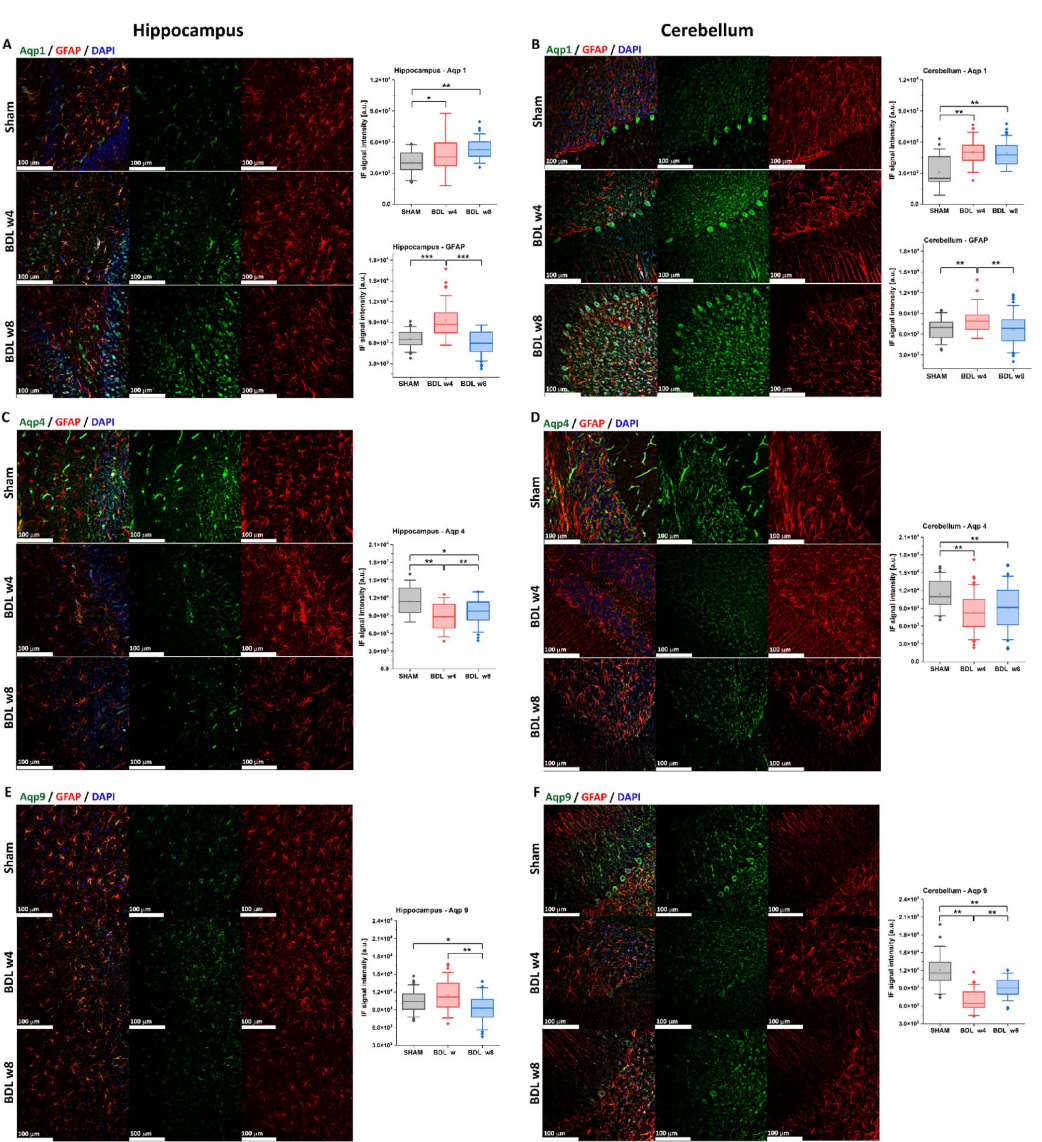
**Representative micrographs showing immunostaining of Aqp1, Aqp4, Aqp9 and GFAP** in the hippocampus (left panel) and cerebellum (right panel) of BDL rats at week 4 and 8 post-surgery, and the control SHAM rats. Significant increase of immunolabeling with Aqp1 in BDL rats was visualized in the hippocampus (**A**) and cerebellum (**B**) and confirmed by the quantitative analyses in terms of the fluorescence intensity. Aqp4 expression visualized in vessels and astrocytic endfeet in SHAM rats in the hippocampus (**C**) and cerebellum (**D**) was significantly decreased in BDL rats, confirmed by the decrease of fluorescence intensity. Aqp9 expression decreased significantly in the hippocampal astrocytes (**E**) and the cerebellar Purkinje cells (soma and dendrites) and astrocytes (**F**), confirmed by the decrease of fluorescence intensity. GFAP—the quantitative analyses of the fluorescence intensity in the hippocampus (**A**) and cerebellum (**B**) showed an increase of the GFAP signal in BDL rats at week 4 and the overall decrease at week 8 corroborating with the immunolabeling. Data are presented as mean ± SD and statistical significance: *p < 0.05, **p < 0.01, ***p < 0.001(One-way Anova with post-hoc Tukey HSD). (For interpretation of the references to color in this figure legend, the reader is referred to the Web version of this article.)

Aqp1 immunoreactivity was observed in the hippocampal astrocytes, granular neurons and vessels of SHAM rat’s (Fig. 5A). In the cerebellum of SHAM rats strong Aqp1 expression was seen in the soma of Purkinje cells, and was weaker in the granular cells layer and astrocytes (Fig. 5B). Increased expression of Aqp1 was observed in both brain regions as disease progressed. In the hippocampus, Aqp1 channels were localized to granule cells as well as to astrocytes, specifically within the soma and processes of the latter (Fig. 5A). Quantitative analysis of Aqp1 immunofluorescence signals showed significant increase already at week 4 post-BDL. The immunofluorescence of Aqp1 increased significantly in the hippocampus (+ 32%, *p* < 0.05) and cerebellum (+ 66%, *p* < 0.01) as early as week 4 post-BDL, and continued to rise until week 8 (Fig. 5A,B).

In the SHAM rats the Aqp4 immunostaining was mostly detected around the hippocampal (Fig. 5C) and cerebellar microvessels (Fig. 5D). With the disease progression Aqp4 microvessels immunolabeling decreased significantly in both the hippocampus and cerebellum. At week 4 post-BDL Aqp4 labeling was more visible in the astrocytes processes and endfeet near the blood vessels in the hippocampus (Fig. 5C) but not in the cerebellum. Quantitative analysis revealed a significant decrease in Aqp4 immunofluorescence intensity starting at week 4 post-BDL, with reductions of -32% in the hippocampus (*p* < 0.01) and − 33% in the cerebellum (*p* < 0.01) (Fig. 5C,D).

Aqp9 immunoreactivity was seen in astrocytic somata and processes of the hippocampus and cerebellum of SHAM rats (Fig. 5E,F). In addition, in the cerebellum of the SHAM rat’s Aqp9 immunolabeling was also present in the Purkinje cells, both in soma and in the dendritic trees. The quantitative analysis of Aqp9 immunofluorescence signals in the hippocampus revealed a non-significant signal increase at week 4 post-BDL (+ 9%) followed by a significant decrease at week 8 (− 20%, *p* < 0.01). In the cerebellum the Aqp9 immunolabeling of Purkinje cells was almost lost and the immunofluorescence signals decreased starting at week 4 post BDL (− 33%, *p* < 0.01) (Fig. 5E,F).

As previously reported^19^, GFAP staining revealed a reactive astrocytic response (astrocytosis), characterized by a visible increase in GFAP+ cells at week 4 post-BDL, followed by a reduction in astrocytosis at week 8 compared to week 4 post-BDL (Fig. 5A–E). These findings are consistent with the quantitative analyses of GFAP fluorescence intensity in the hippocampus and cerebellum (Fig. 5A,B), which show an increased GFAP signal in BDL rats at week 4 (hippocampus + 26% (*p* < 0.001), cerebellum + 24% (*p* < 0.01)) and an overall decrease at week 8 vs. week 4 (hippocampus − 55% (*p* < 0.001), cerebellum − 23% (*p* < 0.01)) (Fig. 5A,B).

#### Determination of neurodegeneration markers in blood

##### NfL

**Fig. 6:**
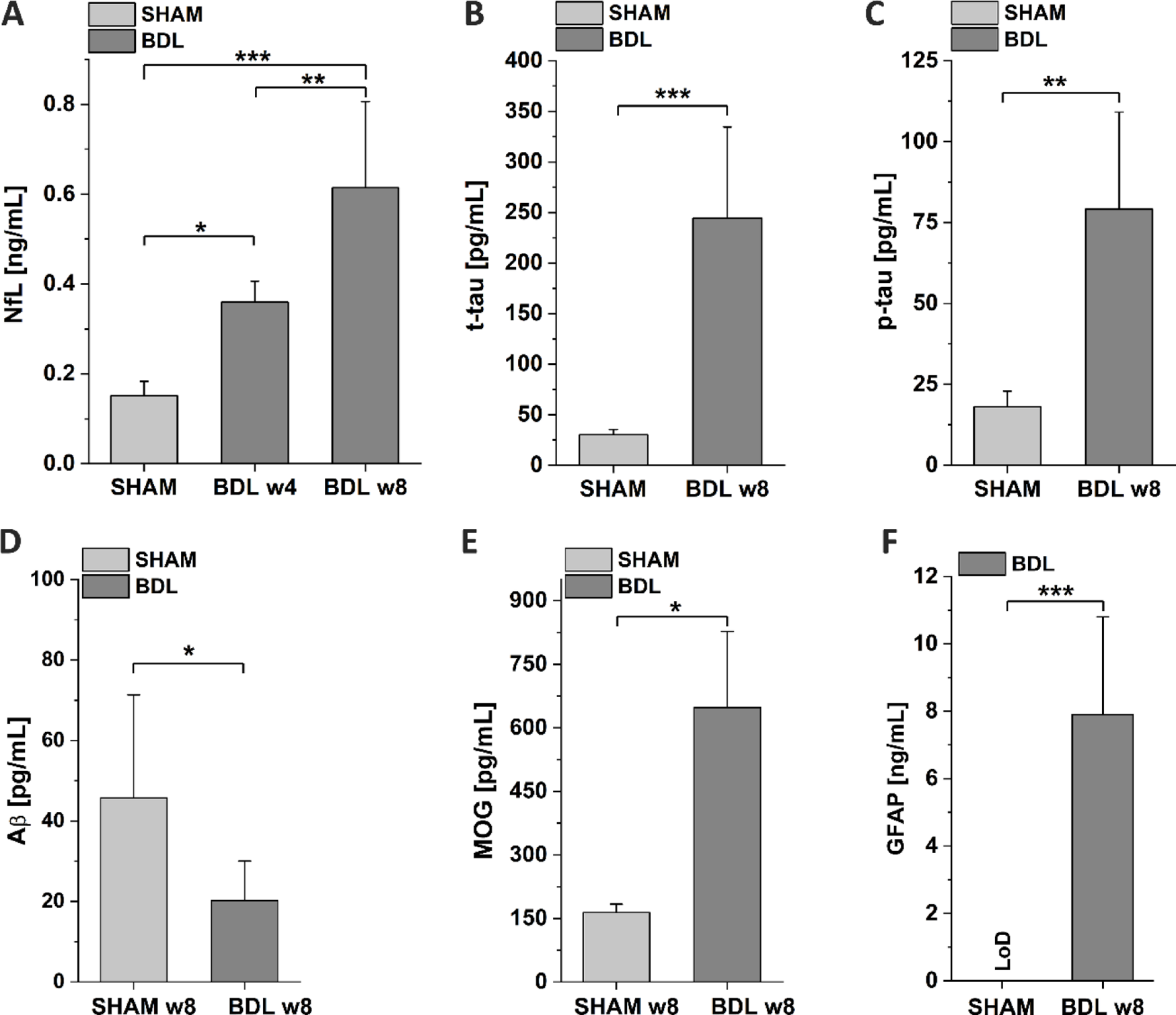
**Blood neurodegeneration markers quantification by ELISA assays. **Comparison of plasma levels of (**A**) NfL, (**B**,**C**) tau-bodies, (**D**) Amyloid β_42_, (**E**) MOG, and (**F**) GFAP between BDL and SHAM rats. Plasma NfL, tau-bodies, MOG, and GFAP levels were higher in the BDL group compared with SHAM controls, while amyloid β concentration decreased, all following the trends observed in Alzheimer Disease. Data are presented as mean ± SD and statistical significance: *p < 0.05, **p < 0.01, ***p < 0.001 (One-way Anova with post-hoc Tukey HSD).

Neurofilament light chain concentrations were measured in plasma of BDL and SHAM rats since its increase is related to axonal damage and is associated to neuroinflammation^20^. The BDL group exhibited significantly higher levels of NfL (+ 137%, *p* < 0.05) compared to the SHAM group as of 4 weeks post BDL surgery and continued to increase until the week 8 (+ 305%, *p* < 0.001) (Fig. 6A).

##### Tau-bodies

Concentrations of tau protein, the axonal cytoskeleton-stabilizing element^21^, was assessed in plasma samples of BDL and SHAM rats. The mean levels of tau proteins were significantly elevated in BDL group at week 8 compared to the SHAM rats (t-tau + 713% (*p* < 0.001) and p-tau + 339% (*p* < 0.01)). Reduced p/t-tau ratio was observed in BDL rats (SHAM: 0.6 vs. BDL: 0.32) (Fig. 6B,C).

##### Amyloid β

The mean plasma levels of Aβ_42_ were significantly decreased in BDL rats at week 8 compared to SHAM group (− 56%, *p* < 0.05). In addition, the Aβ_42_/p-tau and Aβ_42_/t-tau ratios were lower in BDL group compared to SHAM rats, 0.26 vs. 2.53 and 0.08 vs. 1.52, respectively (Fig. 6D).

##### MOG

In case of neuronal damage, the myelin proteins are released into the bloodstream. The concentration of myelin oligodendrocyte glycoprotein in BDL rat plasma at week 8 was significantly higher (+ 295%, *p* < 0.05) than in the SHAM group (Fig. 6E).

##### GFAP

Glial fibrillary acidic protein, a proxy of astrocyte reactivity, was quantified in plasma of BDL and SHAM rats. A significant increase of GFAP was observed in BDL rats at week 8 (*p* < 0.001) compared to the SHAM group where concentrations were below detection limit and attributed to the hypothetical value of 0 (Fig. 6F).

#### Plasma bile acids

**Fig. 7:**
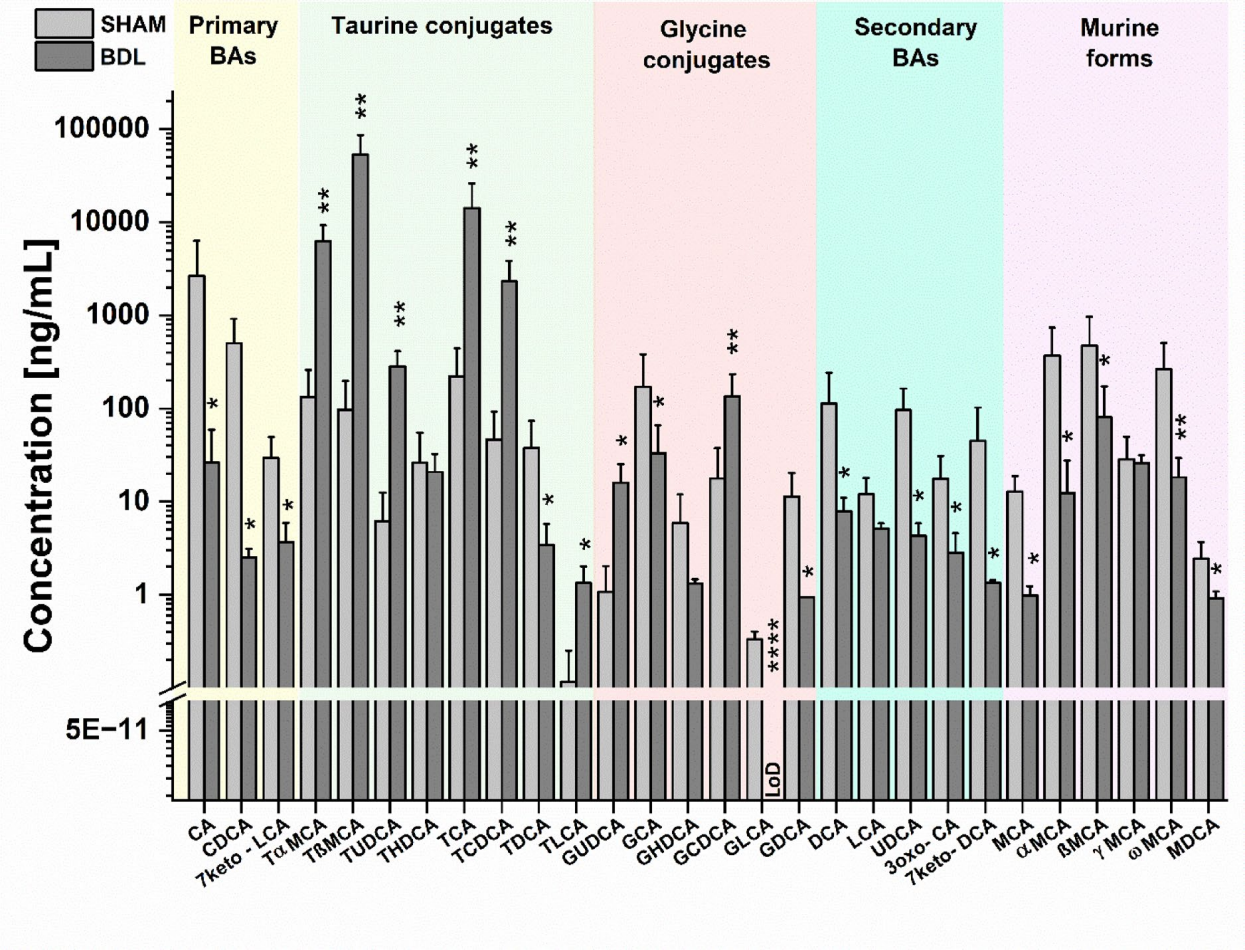
**Comparison of plasma bile acids profiles of SHAM and BDL rats.** Data are presented as mean ± SD and statistical significance: *p < 0.05, **p < 0.01, ***p < 0.001, ****p < 0.0001 (One-way Anova with post-hoc Tukey HSD).

BDL animals exhibited alterations in the bile acids profile (Fig. 7). A significant decrease of primary (including the murine forms) and secondary bile acids was seen. The pool of primary BAs decreased from 3198 to 32.5 ng/mL. The pool of murine forms of primary BAs decreased from 1154 to 139 ng/mL. The pool of secondary BAs decreased from 296.4 to 21.4 ng/mL. However, a significant increase of conjugated bile acids was observed. In particular, the taurine (tauro-α-muricholic acid (TαMCA), tauro-β-muricholic acid (TβMCA), tauroursodeoxycholic acid (TUDCA), taurochenodeoxycholic acid (TCDCA), and taurocholic acid (TCA)) conjugated bile acids caused an increase of total pool of BAs from 5424 to 76,514 ng/mL. In addition, an increased ratio of deoxycholic acid (DCA)/cholic acid (CA) (SHAM: 0.042 vs. BDL: 0.297, sevenfold change) was observed.

## Discussion

This study demonstrates for the first time that chronic liver dysfunction can trigger Alzheimer’s disease-related neuropathological hallmarks. By integrating histological, spectroscopic, and blood-based analyses, we show that BDL rats exhibit central and systemic markers of neurodegeneration compared with SHAM controls. These findings highlight the importance of assessing hepatic function in dementia diagnosis and support a systemic perspective on AD pathogenesis.

In AD, the intraneuronal accumulation of Aβ (iAβ) has been a subject of scientific debate since the 1980s and has been suggested to precede the formation of neurofibrillary tangles and the deposition of extracellular Aβ^22–24^. In the present study, for the first time in CLD rat model an intraneuronal accumulation of Aβ was observed in several brain regions together with tau-bodies in the pre-tangle and NFTs status, preceding extracellular plaque deposition, which aligns with early AD stages. Intraneuronal Aβ accumulation occurred concomitantly with neurofibrillary tangles formation in a time-dependent manner, increasing with disease progression.

Studies on transgenic mice (3xTg-AD mice) suggest that intraneuronal accumulation of Aβ plays a crucial role in triggering cognitive impairment, rather than its extracellular deposition, and correlates with deficits in synaptic transmission and long-term potentiation (LTP)^25,26^. Furthermore, the extracellular deposition occurs in the later stages of the disease and partly originates from iAβ in degenerating neurons^26^. Therefore, neurotoxicity initially attributed to extracellular Aβ may, in fact, represents a downstream consequence of earlier iAβ-mediated damage. Since adult BDL animals have a maximum lifespan of approximately 8 weeks post-surgery^16^, it is likely that the late-stage effects of extracellular Aβ accumulation, hallmarks of advanced AD, were not observed in our model.

The detected cerebral amyloid angiopathy (CAA) is likely due to impaired clearance of Aβ across the blood–brain barrier (BBB), suggesting a failure in the perivascular drainage of soluble Aβ from the brain^27^. This dysfunction weakens the blood vessels and increases the risk of intracerebral hemorrhage. It is thought to contribute to decreased cerebral blood flow, often present in type C HE^28^, and is associated with an increased risk of cognitive impairment^29^. In addition, studies have revealed that Aβ causes vascular dysfunction via oxidative stress^30^.

Oxidative stress is a major pathway driving neurodegeneration^10,12,31^. Neurons are highly sensitive to oxidative stress due to their high oxygen demand, relatively low antioxidant levels, the presence of high concentrations of unsaturated lipids, and metal ions^10^. The brain of both HE and AD patients as well as the brain of the CLD rat model, present significant extent of oxidative damage^10,12,31^. Moreover, in AD, oxidative damage has been associated with accumulation of Aβ and neurofibrillary tangles formation (aggregation and hyperphosphorylation of the tau protein)^32^, something depicted also in the CLD rat model herein.

Aqp1, Aqp4, and Aqp9 are water channel proteins that maintain water balance and buffer extracellular ion concentrations. They have been identified in the mammalian brain and are produced under both healthy and pathological conditions^33–35^. Aquaporins also participate in various ancillary functions, such as glutamate clearance in tripartite synapses, neuronal excitation, neurotransmission, signal transduction, neurogenesis, and brain energy metabolism^35^. Additionally, they play a role in cell adhesion and migration^35^.

Aqp 1 plays a pivotal role in maintaining water homeostasis^35,36^, driven by the osmotic gradient^37^ and thereby regulating cell volume^35^. Studies have shown increased Aqp1 expression in activated astrocytes in several neurodegenerative diseases, including AD, multiple sclerosis, epilepsy, and ischemia^33^. Quantitative analysis of Aqp1 immunofluorescence in the CLD rat model revealed a significant increase in signal as early as week 4 post-BDL in both studied brain regions, the hippocampus and cerebellum. Aqp1 immunofluorescence continued to increase in both, astrocytes processes and neurons, as the disease progressed. The observed increase in Aqp1 clearly indicates impaired water transport in the brain of the CLD rat model. Of note, studies in transgenic AD mice (3xTg-AD and 5xFAD) have shown a close association between Aqp1 upregulation and Aβ deposition, highlighting the role of Aqp1 in Aβ clearance^33^. Furthermore, elevated Aqp1 expression can improve cell motility and Aβ clearance at amyloid deposition sites, indicating a response to Aβ-induced stress.

Aqp 4 is the primary water channel in the mammalian brain^37,38^ contributing to neuronal excitability by maintaining osmotic balance and regulating the ionic environment around neurons^36^. Its decrease reported herein as early as week 4 post-BDL in both studied brain regions, the hippocampus and cerebellum, might play a protective role against brain swelling following the induction of cytotoxic edema^36^ due to an increased ammonia load. Furthermore, the perivascular loss of Aqp4 has been reported in neurodegenerative and neurovascular disorders^39^. In AD, Aqp4 loss was specifically associated with Aβ plaque formation and tau pathology^37,40^, as also depicted in this study.

Furthermore, studies in rodents with Aqp4 gene deletion revealed an increase in the apparent diffusion coefficient (ADC) of water, which was linked to altered brain fluid transport and an impaired glymphatic system^41–43^. This impairment slowed CSF influx and interstitial efflux, leading to interstitial fluid stagnation and enlargement of the interstitial space, thereby reducing Aβ clearance and resulting in increased Aβ deposition and further plaque formation^40,42,43^. Likewise, a modest elevation in the ADC of water has been observed in patients with type C HE^44^. This is most likely related to the outflow of solutes and waste products, the inflow of neurotoxins (such as ammonia), and the subsequent neuroinflammation and oxidative stress^10,12^.

Aqp9 an aquaglyceroporin, plays a crucial role in brain energy homeostasis as a metabolite channel for energy substrates such as glycerol and monocarboxylates^42,45,46^. Quantitative analysis of Aqp9 immunofluorescence in the CLD rat model revealed a region-specific pattern of Aqp9 expression. A significant decrease in Aqp9 immunofluorescence was observed in the cerebellum as early as week 4 post-BDL, whereas a comparable reduction in the hippocampus emerged only by week 8. This temporal difference suggests that the cerebellum exhibits an earlier vulnerability to metabolic and osmotic stress compared to the hippocampus.

Given Aqp9 role in transporting glycerol and monocarboxylates, key substrates for energy metabolism^42,46^, its early downregulation in the cerebellum may impair neuronal energy supply, potentially contributing to motor coordination deficits commonly associated with HE^47,48^. The later onset of Aqp9 decline in the hippocampus likely disrupts neuronal energy homeostasis at a more advanced stage, corroborating the progressive cognitive impairment/progressive memory deficits seen in CLD and AD^49^.

Furthermore, the downregulation of Aqp9 may impair its role and significantly contribute to alterations in glucose metabolism, as demonstrated in our previous ^18^F-FDG PET studies using the same CLD rat model^18^, and aligns with findings of glucose hypometabolism in patient with decompensated cirrhosis^50^.

Moreover, research using APP transgenic mice (APPswe/PS1dE9) indicated that Aqp9 downregulation could exacerbate Aβ-induced neurotoxicity, facilitate Aβ-mediated Alzheimer’s disease progression, disrupt synaptic function, and promote apoptosis^46^,thus, indicating the key role of Aqp9 in preserving neuronal vitality.

Glial fibrillary acidic protein is the main intermediary filament of astrocytes, the most abundant cell type in the CNS^51,52^. GFAP plays a central role in maintaining astrocyte morphology, myelination, and the integrity of the blood–brain barrier^53^.

In the present study, GFAP immunolabeling followed by UV–Vis spectroscopy of immunofluorescence revealed a statistically significant increase in signal at week 4 post-BDL surgery, followed by a significant decrease at week 8 compared to week 4. These results corroborate our previous findings, which demonstrated cytoskeletal alterations in astrocytes in the CLD rat model, characterized by a decrease in processes and the number of intersections, along with changes in astrocyte numbers over disease progression^11,17,19^. This is a hallmark of astrocyte reactivity and a common feature of neuropathological disorders^54^.

The loss of GFAP-positive cells and glial filaments may result from elevated brain ammonia, which is detoxified in astrocytes through glutamine synthesis, leading to osmotic and oxidative stress and accompanied by neuroinflammation (e.g., IL-6 accumulation in the CNS)^10–12,19^. Additionally, previously reported RNA oxidation can impair translation and gene expression, while oxidative stress may trigger protein depolymerization^10,12^. Because the cytoskeleton relies on an interconnected filament network stabilized by cross-linking proteins, damage to a single component can destabilize the entire structure, promoting OS-driven cytoskeletal breakdown and ultimately astrocytic cell death^10^.

Furthermore, in healthy individuals, GFAP blood levels are generally very low and remain below the detection limits^53^, as observed in SHAM group in this study. The significant increase in GFAP levels in the blood of BDL rats at week 8, as depicted here, aligns with a recent study on GFAP blood concentrations in cirrhotic patients^55^. This finding corroborates the previously observed astrocyte morphological alterations (injury) and activation in BDL rats^11,19^, leading then to the observed increase of serum GFAP concentrations.

Alterations in GFAP expression and the loss of filaments (depolymerization) may play a major role in synaptic loss and changes in function^10,56^. These changes have been shown to closely correlate with reactive astrocytosis and elevated GFAP levels in both CSF and blood, and the density of neuritic plaques in the AD brain^56,57^. This further strengthens the argument for astrocytic activation as a mediator linking amyloid and tau pathology.

NfL is an axonal cytoskeleton component ^20^ that is mostly found in large myelinated axons. It is required for radial growth, structural integrity, and effective nerve impulse transmission^58^. Elevated NfL levels in bodily fluids (CSF and blood) have been linked to brain damage and atrophy^59^ and correlated significantly with cognitive function impairment, particularly in those with neurological disorders^60,61^. Therefore, an increase of NfL levels in plasma of CLD rat model already at 4 weeks post BDL surgery is a clear sign of neuroaxonal damage. Furthermore, recent studies have reported a significant relationship between blood NfL levels and brain atrophy in AD pathology, with elevated concentrations detectable even in the preclinical stages of the disease^59^.

Tau’s principal physiological role is to maintain microtubules within neuronal axons^62^. The neurodegenerative processes leads to neurofibrillary tangles formation and increased release of tau into the CSF and bloodstream^63^, something observed here in the rat model of CLD. Studies have shown a significant relationship between plasma p-tau concentration, cognitive decline, brain atrophy, and glucose hypometabolism^64,65^. Eelevated p-tau levels in patients with subjective cognitive decline (SCD) and mild cognitive impairment (MCI) strongly predicted their progression to AD^66^. Furthermore, a reduced p/t-tau ratio, as observed in this study, has been shown to be a strong predictor of future AD^67^.

Amyloid β is the main component of the amyloid plaques found in AD brains^68^. Studies have shown reduced Aβ_42_ levels in the plasma of AD patients^68^. Similarly, a decrease in Aβ_42_ levels was observed in this study in CLD rat model. It has also been reported that the Aβ_42_/p-tau and Aβ_42_/t-tau ratios significantly decrease in the CSF of AD patients^69^. Consistently, decreased Aβ_42_/p-tau and Aβ_42_/t-tau ratios were observed in the CLD rat model in this study.

Myelin degeneration is believed to contribute to neuronal dysfunction and is associated with Aβ plaque accumulation and tau hyperphosphorylation, ultimately leading to cognitive decline^70^. Myelin oligodendrocyte glycoprotein is an immunoglobulin (autoantigen)^71^ expressed exclusively in CNS, located in the outermost layer of the myelin membrane^72^ being a marker of oligodendrocytes maturity and possibly playing a role in cell adhesion and microtubule stability^71^. The elevated MOG levels observed in BDL rats may indicate oligodendrocyte membrane damage, resulting in a higher efflux of MOG into the bloodstream.

Bile acids play a crucial role in CNS physiology, and alterations in bile acid profiles have been associated with neurodegenerative disorders^73^. Under physiological conditions, the bile acids pool is carefully controlled^74^. An increase in the total bile acid pool concentration is directly linked to liver damage^74^. A reduction in primary bile acids, as observed here, have been linked to gut microbiota imbalance, impaired lipid digestion, and disrupted enterohepatic circulation^10,73^. A 100-fold decrease of primary and tenfold decrease of secondary bile acids in BDL rats resulted in an elevated deoxycholic acid (DCA) to cholic acid (CA) ratio, indicative of the 7α-dehydroxylation of CA by gut bacteria. Studies have shown that an increased DCA/CA ratio is associated with cognitive decline, indicating a potential role of gut-liver-brain axis in the pathogenesis of neurodegenerative diseases^4^. Recent studies support that gut microbiota influences pathological features of Alzheimer’s disease, including neuroinflammation and Aβ accumulation^73^.

In addition, the enteric nervous system connects the gut microbiota with the CNS via vagal signaling, forming a key pathway of the gut-liver-brain axis^10^. As a result, bacterial infections / microbiota alterations and systemic inflammatory responses can alter brain function by stimulating afferent vagal pathways, a process driven by the release of cytokines and chemokines at sites of inflammation, which may subsequently impair cognitive and motor performance^10,75^.

Furthermore, the increased concentration of taurochenodeoxycholic acid (TCDCA), as reported here, has been associated with a decreased mitochondrial membrane potential^73^, a hallmark of mitochondrial disfunction that may lead to loss of cell viability and contribute to various pathologies^76^. In particular, association between bile acids and oxidative stress have been well demonstrated^77^. The release of ROS may result in depletion of antioxidants, oxidation of thiol groups and lipid peroxidation^10,77^.

## Conclusions

This is the first study to our knowledge to demonstrate a direct association between liver function and the risk of Alzheimer’s disease. Our results provide further evidence of liver implication in Aβ and tau accumulation. Although these associations are robust, the underlying mechanisms still need to be clarified.

Our findings offer valuable insights for future research into the link between liver function and AD pathogenesis, as well as for developing prevention and treatment strategies that address liver dysfunction. In particular, our work introduces a novel preclinical paradigm that bridges hepatology and neurology, shifting the conventional brain-centric view of AD toward a systemic disease model.

Importantly, these results highlight the need for a more holistic and physiological approach to AD and other dementias, considering patient comorbidities and adopting a multidisciplinary strategy to study the pathobiology of AD and other dementias.

## Materials and methods’

### Animal welfare

In line with the 3R (Replacement, Reduction, and Refinement) principles, the rats used in the current study have been previously described and characterized in earlier publications ^11‚19^. In the current study a range of additional histological stainings and blood analyses were conducted. As such, no additional animals were euthanized for this work. All procedures were done in accordance with the animal care and were approved by The Committee on Animal Experimentation for the Canton de Vaud, Switzerland (VD3022) and complied with the ARRIVE guidelines.

### Chronic liver disease model

Wistar male adult rats (BDL n = 31, SHAM n = 18, Charles River Laboratories, L’Arbresle, France) were group-housed in the animal facilities of CIBM-AIT in Lausanne for BDL experiments. The BDL model was selected to represent chronic liver disease (CLD) because it reliably induces cholestatic liver injury and fibrosis within a predictable timeframe^16^. Animals underwent bile-duct ligation (BDL—recordings of surgery steps: https://zenodo.org/records/10652104) for CLD-induced HE, recognized by the International Society for Hepatic Encephalopathy and Nitrogen Metabolism (ISHEN^16^), and sham surgery. The Supplementary Material & Methods contains a detailed information about the number of animals used for each experiment (Table S1).

### Biochemical measurements

Chronic liver disease was confirmed by the early increase in plasma ammonia and bilirubin at 2 weeks post-BDL. Liver parameters were monitored longitudinally at weeks 0, 2, 4, 6, and 8. Plasma bilirubin, aspartate aminotransferase (AST/GOT), and alanine aminotransferase (ALT/GPT) were measured using the Reflotron® Plus system (F. Hoffmann-La Roche Ltd), blood ammonia was assessed with a blood ammonia meter (PocketChem™ BA PA-4140), and blood glucose levels were determined using the Contour XT (Bayer, Germany), as previously reported^12^.

Blood samples (BDL n = 31, SHAM n = 18) were taken from the sublingual vein into anti-coagulated tubes (EDTA). Blood was collected in pre-cooled K3EDTA tubes (Sarstedt AG & Co. KG), centrifuged at 2,700 × g for 7 min at 4 °C, and stored at − 80 °C until the assay procedure.

### Histology

Rats were pre-anesthetized with 4% isoflurane (Piramal Enterprises Ltd.) for 5 min, followed by a subcutaneous injection of the analgesic Temgesic (ESSEX) at 0.03 mg/mL in 0.9% NaCl. Fifteen minutes later, an intracardiac perfusion was performed via the left ventricle using phosphate-buffered saline (PBS, P5493 Sigma, pH 7.4). Following perfusion, the animals were euthanized by decapitation. The brains were then removed, fixed in 4% formaldehyde in PBS overnight at 4 °C, washed with PBS, and embedded in paraffin. The brains were sectioned into 8 µm thick sagittal sections for standard histochemistry (4-weeks (n = 3) and 8-weeks (n = 3) post-BDL and SHAM (n = 3) surgery). For immunohistochemistry after 4% formaldehyde fixation brains were cryopreserved in 30% sucrose PBS solution at 4 °C for 48 h, and then embedded in Tissue-Tek O.C.T. compound and then cut into 16 µm sagittal-sections (4-weeks (n = 3) and 8-weeks (n = 3) post-BDL and SHAM (n = 3) surgery). For both techniques a 7 slides/rat were used (histochemistry: 126 slides prepared, immunohistochemistry: 189 slides prepared).

The selected 4- and 8-week (humane endpoint) intervals allow for longitudinal assessment of histological changes during the progression of chronic liver disease.

### Histochemistry

#### Amyloid β pathology

Congo Red staining (CR protocol, IHC WORLD, LLC) was used to determine Amyloid pathology https://ihcworld.com/2024/01/26/modified-high-ph-congo-red-staining-protocol-for-amyloid/.

#### Tau protein pathology

The Gallyas Silver Stain technique was used to investigate tau protein pathology according to the protocol described in GS protocol https://www.protocolsonline.com/histology/dyes-and-stains/neurohistology/gallyas-silver-stain/ .

For both staining’s slides were deparaffinized and rehydrated in distilled water before being incubated in working solution and washed in distilled water. Finally, they were differentiated and then dehydrated through a graded series of ethanol’s, washed in xylene, and mounted in resinous mounting media.

Tissue sections were examined using bright field microscopy with a MEIJI-TECHNO TC5600 microscope. Images were captured and processed using INFINITY ANALYZE 7 software (Lumenera, Canada).

### Immunohistochemistry and immunofluorescence (UV–Vis spectroscopy)

Two brain regions were selected for immunohistochemical analysis: the hippocampus and the cerebellum. The hippocampus was examined as it is one of the earliest structures affected in Alzheimer’s disease and is critically involved in cognitive and non-cognitive functions. The cerebellum was included due to its essential role in the regulation and maintenance of these processes^78,79^. Three sets of tissue sections were incubated with a polyclonal rabbit anti-aquaporin antibody: Aqp1 (PA5-77842, Thermo Fisher) (2 h at RT) at 1/100 dilution, Aqp4 C-terminus (PA5-77716, Thermo Fisher) (2 h at RT) at 1/200 dilution, Aqp9 (PA5-114872, Thermo Fisher) (2 h at RT) at 1/100 dilution with Alexa Fluor® Plus 488 secondary goat anti rabbit antibody IgG (H + L, A32731, Thermo Fisher) (1 h at RT) at 1/1000 dilution and then counterstained with mouse monoclonal anti-GFAP antibody (glia-specific intermediate-filament protein, MAB360, Merck Millipore)(2 h at RT) at 1/100 dilution with secondary Alexa Fluor® 594-AffiniPure rat anti-mouse IgG (H + L) antibody (415-585-166, Jackson ImmunoResearch Europe Ltd.) (1 h at RT) at 1/200 dilution were used to visualize astrocytes. Nuclei were stained with DAPI (D1306, Thermo Fisher).

The Leica THUNDER Imaging System was integrated with the Ocean HDX UV–Vis spectrometer (Ocean Insight, Florida, USA) and the Lab Grade Reflection Probe R400-7-UV–VIS. Fluorophore signals were captured using excitation, dichroic, and emission filter sets (Chroma Technology Corporation: EM: ET610Ip, BS: ZT594rdc, EX: ET580/25) to detect fluorescence signals. The total Aqp1, Aqp4, Aqp9, and GFAP signal intensities were quantified by integrating the UV–Vis spectra. Spectra were evaluated and processed by OriginPro (OriginLab, USA).

### Determination of blood neurodegeneration markers

#### Neurofilament quantification

Quantitative determination of neurofilament light chain (NfL) levels was performed in plasma samples from BDL (4-weeks n = 6 and 8-weeks n = 10) and SHAM (n = 4) rats. Plasma NfL levels were measured in duplicate using ELISA NfL kit, according to the manufacturer’s instructions and standard procedures (CUSABIO: CSB-EL015688RA, analytical sensitivity: 1.95 pg/mL, detection range: 7.8–500 pg/mL).

#### Phosphorylated p-tau and total t-tau quantification

The levels of p-tau and t-tau were determined in plasma samples from BDL (8-weeks n = 4) and SHAM (n = 3) rats. Plasma p-tau and t-tau levels were measured in duplicate using ELISA p-tau (AssayGenie: RTFI01098, analytical sensitivity: 9.375 pg/mL, detection range: 15.625–1000 pg/mL) and t- tau kits (AssayGenie: RTFI00944, analytical sensitivity: 18.75 pg/mL, detection range: 31.25–2000 pg/mL), according to the manufacturer’s instructions and standard procedures.

#### Amyloid β quantification

Quantitative determination of amyloid β (Aβ) levels, was performed in plasma samples from BDL (8-weeks n = 5) and SHAM (n = 6) rats. Plasma Aβ levels were measured in duplicate using ELISA Aβ kit (Invitrogen: KMB3441, analytical sensitivity: < 3 pg/mL, detection range: 3.12–200 pg/mL), according to the manufacturer’s instructions and standard procedures.

#### GFAP quantification

GFAP levels were determined in plasma samples from BDL (8-weeks n = 8) and SHAM (n = 6) rats using the highly sensitive Rat GFAP ELISA Kit (AssayGenie: RTES00998, sensitvity: 0.19 ng/mL, detection range: 0.31–20 ng/mL). Samples were measured in duplicate according to the manufacturer’s instructions.

#### Myelin oligodendrocyte glycoprotein (MOG) quantification

The levels of MOG were determined in plasma samples from adult (8-weeks n = 9) and SHAM (n = 4) rats. Plasma MOG levels were measured in duplicate using ELISA MOG (AVIVA SYSTEMS BIOLOGY: OKCD00239, analytical sensitivity: 0.055 ng/mL, detection range: 0.156–10 ng/mL) kit, according to the manufacturer’s instructions and standard procedures.

The absorbance was measured at 450 nm with microplate reader Hidex Sense Beta (Hidex Oy).

### Plasma bile acids

Isotope-dilution high performance liquid chromatography coupled to high resolution mass spectrometry (LC–MS) was used^80^ to measure the bile acids at 8 weeks after BDL (n = 7) and SHAM (n = 8) surgery. The experiment was carried out by mixing the specimen (50 μL) with the internal standards in methanol (100 μL) and then adding 600 μL of H_2_O containing 0.2% formic acid. HLB SPE plates (Waters) were used for sample extraction and cleaning. Thermo Q-Exactive (Thermo Fisher Scientific) was used for LC–MS analysis. A 10 μL extract volume was injected into an Acquity UPLC HSS T3 1.8 μm, 2.1 100 mm column. The MS was set to full-scan acquisition in negative mode (-H m/z 370 to 522, centroid acquisition, negative polarity).

### Statistical analysis

For ELISA and UV–Vis spectroscopy one-way ANOVA followed by post-hoc Turkey HSD was used to compare BDL and SHAM-operated rats, except for the GFAP and GLCA bile acid level in the blood, which were analyzed using a one-sample Student’s t-test against a hypothetical value of 0, as the levels in SHAM (GFAP) and BDL (GLCA bile acid) rats were below the detection limit. All tests were 2-tailed, Significance level in all tests was attributed as follows: *p < 0.05, **p < 0.01, ***p < 0.001, ****p < 0.0001. Statistical analyses were performed using OriginPro (OriginLab, USA).

#### Study limitations

One of the limitations of this study is the exclusive use of male rats, which may limit the generalizability of the findings to both sexes, as sex differences in liver disease progression and AD vulnerability may affect outcomes. More work with both male and female rats is necessary to determine whether these results are consistent across sexes. Additionally, the absence of behavioral assessments is a limitation of the present study, as such measures could provide important insights into functional consequences of the observed changes. To address these issues, we have now initiated experiments using female rats to explore potential sex-specific differences.

## Supplementary Information

Supplementary Information.

## Data Availability

The data used to support the findings of this study are available from the corresponding author upon request.

## Abbreviations

Aβ: Amyloid beta
Aqp: Aquaporin
ALT/GPT: Alanine aminotransferase
AST/GOT: Aspartate aminotransferase
BBB: Blood-brain barrier
BDL: Bile duct ligation
CAA: Cerebral amyloid angiopathy
CLD: Chronic liver disease
CNS: Central nervous system
GFAP: Glial fibrillary acidic protein
MOG: Myelin oligodendrocyte glycoprotein
NfL: Neurofilament light chain
IHC: Immunohistochemistry
type C HE: Type C hepatic encephalopathy
