# Supplementary material for: Liver dysfunction triggers early Alzheimer’s pathology in an adult rat model of chronic liver disease
Source: Sci Rep. 2025 Oct 30;15:37953. doi: 10.1038/s41598-025-21054-x (PMC12575788; doi:10.1038/s41598-025-21054-x)
Supplement: Supplementary file 1 — Supplementary Information. [file 41598_2025_21054_MOESM1_ESM.docx]

# **Supplementary Materials**

**Table S1. Detailed report on each rat used in the study**

| **SHAM rat No.** | **Blood biochemistry** | **ELISA** | | | | | | **Histochemistry**  **AB & Gallyas** | **IHC +UV-Vis**  **Aqp's & GFAP** | **Bile Acids** |
| --- | --- | --- | --- | --- | --- | --- | --- | --- | --- | --- |
|  |  | **NfL** | **t-tau** | **p-tau** | **Aβ** | **MOG** | **GFAP** |  |  |  |
| **239** | **x** |  |  |  |  |  |  |  |  | **x** |
| **240** | **x** |  |  |  |  |  |  |  |  | **x** |
| **w0 322** | **x** |  |  |  |  |  | **x** |  |  |  |
| **w0 323** | **x** |  |  |  |  |  | **x** |  |  |  |
| **388** | **x** | **x** | **x** | **x** |  |  |  |  | **x** |  |
| **451** | **x** | **x** | **x** | **x** |  |  |  |  |  | **x** |
| **469** | **x** | **x** |  |  | **x** |  | **x** | **x** | **x** |  |
| **470** | **x** | **x** | **x** | **x** | **x** |  | **x** | **x** | **x** |  |
| **w0 472** | **x** |  |  |  | **x** |  |  |  |  |  |
| **489** | **x** |  |  |  |  |  |  | **x** |  |  |
| **526** | **x** |  |  |  | **x** | **x** |  |  |  |  |
| **527** | **x** |  |  |  | **x** | **x** |  |  |  |  |
| **528** | **x** |  |  |  | **x** | **x** |  |  |  |  |
| **536** | **x** |  |  |  |  | **x** | **x** |  |  | **x** |
| **537** | **x** |  |  |  |  |  | **x** |  |  | **x** |
| **548** | **x** |  |  |  |  |  |  |  |  | **x** |
| **549** | **x** |  |  |  |  |  |  |  |  | **x** |
| **550** | **x** |  |  |  |  |  |  |  |  | **x** |
| **TOTAL** | **n=18** | **n=4** | **n=3** | **n=3** | **n=6** | **n=4** | **n=6** | **n=3** | **n=3** | **n=8** |
|  | | | | | | | | | | |
| **BDL rat No.** |  | | | | | | | | | |
| **241** | **x** |  |  |  |  |  |  |  |  | **x** |
| **242** | **x** |  |  |  |  |  |  |  |  | **x** |
| **322** | **x** |  |  |  |  |  | **x** |  |  |  |
| **323** | **x** |  |  |  |  |  | **x** |  |  |  |
| **385** | **x** |  |  |  |  |  | **x** |  |  |  |
| **401** | **x** |  |  |  |  |  | **x** |  |  |  |
| **414** | **x** | **x** |  |  |  |  |  |  |  |  |
| **415** | **x** | **x** |  |  |  |  |  |  |  |  |
| **451** | **x** |  |  |  |  |  |  |  |  | **x** |
| **468** | **x** |  |  |  |  |  |  | **x** | **x** |  |
| **472** | **x** | **x** |  |  |  |  | **x** |  | **x** |  |
| **475** | **x** |  |  |  |  |  |  |  | **x** |  |
| **476** | **x** |  |  |  |  |  |  | **x** | **x** |  |
| **477** | **x** |  |  |  |  |  |  | **x** |  |  |
| **478** | **x** |  |  |  |  |  |  |  | **x** |  |
| **481** | **x** | **x** |  |  | **x** |  |  |  |  |  |
| **482** | **x** | **x** |  |  | **x** |  |  | **x** |  |  |
| **485** | **x** |  |  |  |  |  |  |  | **x** |  |
| **486** | **x** | **x** |  |  | **x** |  |  | **x** |  |  |
| **487** | **x** | **x** |  |  |  |  |  | **x** |  |  |
| **529** | **x** |  |  |  | **x** | **x** |  |  |  |  |
| **530** | **x** |  |  |  |  | **x** |  |  |  |  |
| **531** | **x** |  |  |  |  | **x** |  |  |  |  |
| **532** | **x** |  |  |  | **x** | **x** | **x** |  |  | **x** |
| **533** | **x** |  |  |  |  | **x** |  |  |  | **x** |
| **534** | **x** |  |  |  |  | **x** | **x** |  |  | **x** |
| **535** | **x** |  |  |  |  |  | **x** |  |  | **x** |
| **579** | **x** |  |  | **x** | **x** |  |  |  |  |  |
| **580** | **x** |  |  | **x** | **x** |  |  |  |  |  |
| **602** | **x** |  | **x** | **x** |  |  |  |  |  |  |
| **603** | **x** | **x** | **x** | **x** |  | **x** |  |  |  |  |
| **606** | **x** | **x** | **x** | **x** |  | **x** |  |  |  |  |
| **607** | **x** | **x** | **x** | **x** |  | **x** |  |  |  |  |
| **TOTAL** | **n=33** | **n=10** | **n=4** | **n=6** | **n=7** | **n=9** | **n=8** | **n=6** | **n=6** | **n=7** |

**Congo Red fluorescence:** when bound to β-sheet-rich amyloid fibrils, exhibits a characteristic increase in absorption accompanied by a red shift, as well as enhanced fluorescence. These spectral changes are indicative of its specific interaction with amyloid structures^1-4^.

**
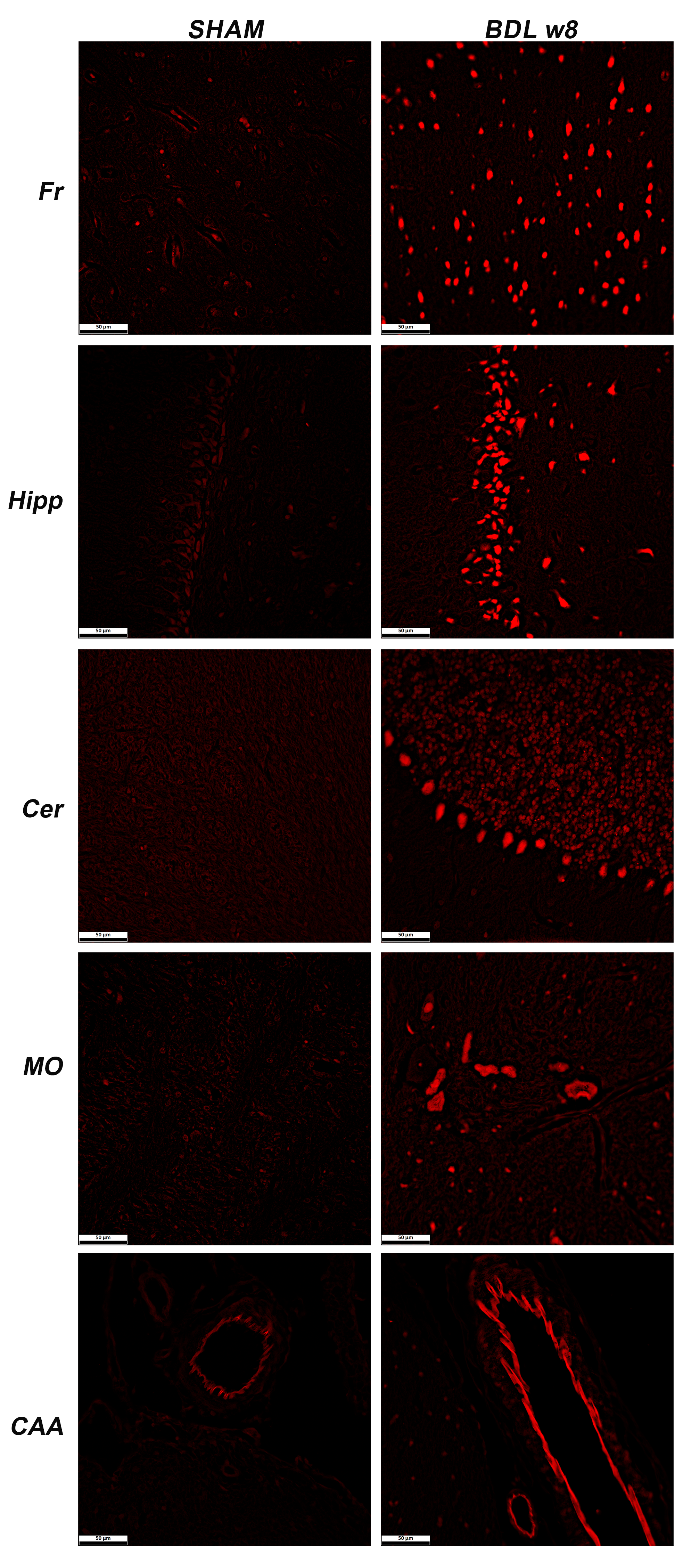
**

**Figure S1.** **Visualization of amyloid deposits in the brain tissue - Congo red fluorescence using a Texas-red filter.** Representative microphotographs of the BDL rats’ brain and age-matched SHAM controls (examples of: frontal cortex (Fr), hippocampus (Hipp), cerebellum (Cer), medulla oblongata (MO), and cerebral amyloid angiopathy (CAA) (scale bar 50μm).

**References**

1 Sen, S. & Basdemir, G. Diagnosis of renal amyloidosis using Congo red fluorescence. *Pathol Int* **53**, 534-538, doi:10.1046/j.1440-1827.2003.01513.x (2003).

2 El-Meanawy, A., Mueller, C. & Iczkowski, K. A. Improving sensitivity of amyloid detection by Congo red stain by using polarizing microscope and avoiding pitfalls. *Diagn Pathol* **14**, 57, doi:10.1186/s13000-019-0822-4 (2019).

3 Linke, R. P. Highly sensitive diagnosis of amyloid and various amyloid syndromes using Congo red fluorescence. *Virchows Arch* **436**, 439-448, doi:10.1007/s004280050471 (2000).

4 Wu, C., Scott, J. & Shea, J. E. Binding of Congo red to amyloid protofibrils of the Alzheimer Abeta(9-40) peptide probed by molecular dynamics simulations. *Biophys J* **103**, 550-557, doi:10.1016/j.bpj.2012.07.008 (2012).
